# Change in obsessive beliefs as predictor and mediator of symptom change during treatment of obsessive-compulsive disorder – a process-outcome study

**DOI:** 10.1186/s12888-016-0914-6

**Published:** 2016-07-07

**Authors:** Alice Diedrich, Philipp Sckopke, Caroline Schwartz, Sandra Schlegl, Bernhard Osen, Christian Stierle, Ulrich Voderholzer

**Affiliations:** Department of Psychiatry and Psychotherapy, University of Munich (LMU), Nußbaumstr. 7, 80336 Munich, Germany; Department of Psychology, University of Munich (LMU), Leopoldstr. 13, 80802 Munich, Germany; Schön Clinic Bad Bramstedt, Birkenweg 10, 24576 Bad Bramstedt, Germany; Schön Clinic Roseneck, Am Roseneck 6, 83209 Prien am Chiemsee, Germany; Department of Psychiatry and Psychotherapy, University of Freiburg, Hauptstr. 5, 79104 Freiburg, Germany

**Keywords:** Obsessive-compulsive disorder, Inpatient treatment, Cognitive behavioral therapy, Obsessive beliefs, Mediator, Change mechanism

## Abstract

**Background:**

Cognitive models of obsessive-compulsive disorder suggest that changes in obsessive beliefs are a key mechanism of treatments for obsessive-compulsive disorder. Thus, in the present process-outcome study, we tested whether changes in obsessive beliefs during a primarily cognitive behavioral inpatient treatment predicted treatment outcome and whether these changes mediated symptom changes over the course of treatment.

**Methods:**

Seventy-one consecutively admitted inpatients with obsessive-compulsive disorder were assessed with the Yale-Brown Obsessive-Compulsive Scale and the Obsessive Beliefs Questionnaire at treatment intake, after six weeks of treatment and at discharge, and with the Beck-Depression-Inventory-II at intake and discharge.

**Results:**

Changes in obsessive beliefs during the first six weeks of treatment predicted obsessive-compulsive symptoms at discharge when controlling for obsessive-compulsive and depressive symptoms at intake in a hierarchical regression analysis. Multilevel mediation analyses showed that reductions in obsessive beliefs partially mediated improvements in obsessive-compulsive symptoms over time.

**Conclusions:**

Our findings indicate that decreasing obsessive beliefs in inpatient cognitive behavioral therapy for obsessive-compulsive disorder might be a promising treatment approach.

## Background

Obsessive-compulsive disorder (OCD) is characterized by a pattern of repetitive obsessive thoughts, images, or impulses and a ritualized pattern of covert mental acts or overt behaviors, aimed at reducing associated negative emotions, including anxiety and fear [1]. According to the International Classification of Diseases-10 (ICD-10), three OCD subtypes exist: one with predominantly obsessional thoughts and ruminations, one with predominantly compulsive acts, and one with mixed obsessional thoughts and acts. OCD is a significant, often chronic [2, 3] and highly comorbid [4] mental health problem. Moreover, it is associated with a reduced quality of life [5], functional impairment [5], and anguish for patients and their families [6]. As such, it constitutes the 10^th^ leading cause of disability among health conditions [7].

Cognitive behavioral therapy (CBT) including exposure and response prevention (ERP) as well as the modification of dysfunctional beliefs about the meaning and significance of obsessive thoughts has been recommended as the treatment of choice in treatment guidelines [8–10] – either alone or in combination with selective serotonin reuptake inhibitors. Several treatment outcome studies have established the (long-term) effectiveness of (C)BT and selective serotonin reuptake inhibitors in both out- and inpatient settings [11–18]. However, prior research also shows that OCD symptoms persist at moderate levels even after an adequate treatment course [3, 11], and some studies indicate that about 30 to 50 % of patients do not benefit from CBT in terms of symptom reduction [11, 19, 20]. Thus, current treatments still need to be further improved. One way to reach this goal is to identify central predictors and mediators of symptom reductions during treatment for OCD [21, 22].

Cognitive behavioral models of OCD emphasize the role of dysfunctional beliefs in the development and maintenance of OCD (see for example, [23–25]). Specifically, they postulate that obsessive beliefs contribute to negative appraisals of intrusive thoughts, i.e. obsessional thoughts, which may then cause compulsive behavior to reduce associated negative emotions. Hence, obsessive beliefs can be considered as predisposing factors of obsessional thoughts and compulsive acts. A number of cognitive models of OCD exist and each of those models focuses on a different belief domain regarding the etiology and maintenance of the disorder [23, 24, 26]. Consistently, the Obsessive Compulsive Cognitions Working Group (OCCWG) [27, 28] identified the following dysfunctional belief domains to be important in this context: inflated sense of personal responsibility, need for certainty, perfectionism, threat estimation, importance of thoughts, and need to control thoughts. Preliminary evidence for the significance of dysfunctional beliefs as a risk and maintaining factor in OCD comes from studies demonstrating positive cross-sectional associations between obsessive beliefs and obsessive-compulsive symptom severity both in non-clinical samples [29, 30] and samples with individuals suffering from OCD [31–34].

Based on this theoretical and empirical background, it might be assumed that the extent of obsessive beliefs at the beginning of treatment impacts treatment outcome. Moreover, it might be hypothesized that changes over the course of treatment predict treatment outcome or even constitute an important mechanism of treatment in OCD. Preliminary evidence for these hypotheses comes from treatment studies indicating that symptom changes during psychotherapy and/or medical treatment are either predicted by the extent of obsessive beliefs at the beginning of treatment [35, 36] or associated with changes in obsessive beliefs during treatment [14, 35, 37–40]. Only one study so far has yielded evidence that changes in maladaptive beliefs precede changes in obsessive-compulsive symptoms during an outpatient cognitive therapy [41]. However, until now, it has never been examined whether treatment-associated changes in obsessive beliefs predict and explain changes in obsessive-compulsive symptoms during an intensive primarily cognitive behavioral inpatient treatment. Therefore, the first aim of the present study was to examine whether changes in obsessive beliefs during the first six weeks of treatment predict treatment outcome in patients suffering from severe OCD. The second aim was to examine the mediating effect of changes in obsessive beliefs on symptom changes during treatment to test whether changes in obsessive beliefs cause changes in treatment outcome.

## Methods

### Participants and design

**Fig. 1:**
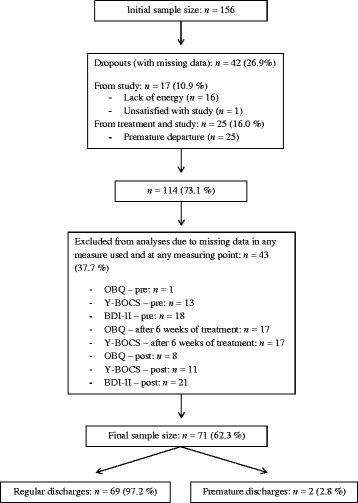
Flow chart of the sample. The described percentages refer to the sample size reported before

The present study was part of another study examining common factors of inpatient psychotherapy of patients suffering from OCD in an uncontrolled repeated measures design (in preparation). One hundred fifty six patients who received an inpatient treatment during January 2011 and May 2013 in two medical centers in Germany were included in the study. To be included, the participants were required to meet the criteria of an obsessive-compulsive disorder according to the Diagnostic and Statistical Manual of Mental Disorders-IV (DSM-IV), to be fluent in German, and to be between 18 and 65 years of age. Participants were excluded if they had a high risk of suicide, an organic brain disorder, or a severe medical condition. To enhance the external validity of the study, we included patients who met criteria for comorbid disorders in addition to OCD, with the exception of patients with a current diagnosis of substance abuse or dependence, psychotic disorder or bipolar disorder. From the initial sample of 156 individuals, 42 participants were not included in the analyses as they were study or treatment drop-outs with missing data. Another 43 patients were excluded due to missing data without having dropped out from study or treatment. Thus, our final sample consisted of *N* = 71 individuals (for patient flow, see Fig. 1; for characteristics of the final sample, see Table 1).

**Table 1:** Sample characteristics

	*M* (*SD*)/% (*N*)		% (*N*)
Female gender	59.2 (42)	Comorbid mental disorders	83.1 (59)
Age (years)	34.59 (11.20)	Depressive disorders	77.5 (55)
High school degree	56.3 (40)	Dysthymic disorder	2.8 (2)
In a relationship	38.0 (27)	Anxiety disorders	18.3 (13)
OCD subtypes		Adjustment disorder	2.8 (2)
F42.0	4.2 (3)	Somatoform disorders	4.2 (3)
F42.1	5.6 (4)	Eating disorders	8.5 (6)
F42.2	90.1 (64)	Personality disorders	9.9 (7)

Note. *N* = 71. *OCD* = Obsessive-Compulsive Disorder. F42.0 = Predominantly obsessional thoughts and ruminations, F42.1 = Predominantly compulsive acts, F42.2 = Mixed obsessional thoughts and acts. Anxiety disorders comprise panic disorder, generalized anxiety disorder, phobic disorder and posttraumatic stress disorder. As all participants were Caucasians we do not report differences with regard to ethnicity. High School = German equivalent of high school certificate after 13 years of schooling

Patients from the initial and the final sample did not differ significantly in terms of demographic (gender, age, education), clinical (type OCD, comorbidities, severity of obsessive-compulsive and depressive symptoms, extent of obsessive beliefs) or treatment-specific (duration, anti-depressive medication) variables (all *p*s > .05) except for relationship (*χ*^*2*^(1) = 4.32, *p* = .04). Individuals who were excluded from the study were more likely to be in a relationship than those who were included in the final sample.

### Procedures

At admission to the hospital, an overall diagnostic assessment according to the criteria of ICD-10 was administered by clinical psychologists or psychiatrists for treatment purposes. For research purposes, DSM-IV diagnoses of OCD were also entered using the German version of the Structural Clinical Interview for DSM-IV [42]. It was conducted by clinical psychologists who had at least a Master’s degree and were enrolled in a PhD-program. The diagnostic assessments were thoroughly discussed with an experienced supervisor (as they were therapists in clinical training). Patients were assessed with the Yale-Brown Obsessive Compulsive Scale (Y-BOCS) [43] and the Obsessive Beliefs Questionnaire (OBQ) [44] at intake, after six weeks of treatment and at discharge, and with the Beck Depression Inventory-II (BDI-II) [45] at treatment intake and discharge. Means and standard deviations of the measures are provided in Table 2.

**Table 2:** Descriptive statistics for measurements at T1, T2, and T3

	T1		T2		T3	
	*M*	*SD*	*M*	*SD*	*M*	*SD*
Y-BOCS	24.82	5.96	19.82	7.37	16.99	7.50
OBQ - Global	4.41	1.17	4.15	1.17	3.80	1.12
OBQ-RES	4.78	1.69	4.55	1.61	4.25	1.59
OBQ-PER	4.87	1.34	4.66	1.30	4.24	1.20
OBQ-IMP	3.37	1.62	3.04	1.54	2.60	1.26
BDI-II	21.76	10.47			14.35	11.47

Note. *N* = 71. T1 = intake, T2 = after six weeks of treatment, T3 = discharge. *Y-BOCS* =  Yale-Brown Obsessive Compulsive Scale. *OBQ - Global = * Obsessive Beliefs Questionnaire Global Score. *RES* =  responsibility and threat estimation. *PER* = perfectionism and intolerance for uncertainty. *IMP* =  importance and control of thoughts (metacognitions). *BDI-II* =  Beck Depression Inventory-II

### Treatment

Patients received a multimodal, intensive inpatient treatment program including both individual and group psychotherapy. Individual therapy took place once or twice a week for 50 min. Group therapies included occupational therapy, music therapy, sports therapy, and a disorder-specific group. Individual therapy and the disorder-specific group were both based on the cognitive behavioral model. They included the following elements: psycho-education about OCD symptoms and the CBT rationale, individualized case formulation (including the identification of potential functions of the symptoms), in vivo ERP and the modification of interpretations of obsessive thoughts as well as of obsessive beliefs. However, the focus of the treatment lay on ERP - both therapist-guided and alone. Treatments were applied by clinical psychologists and/or psychiatrists who were all trained in CBT and supervised by experienced therapists. Most patients (84.5 %) took antidepressants during treatment. Mean treatment duration was 65.41 days (*SD* = 24.15). Almost all patients of the final sample (97.2 %) were discharged regularly.

### Measures

#### Yale-Brown Obsessive Compulsive Scale

The German version of the Y-BOCS [43, 46] was used to measure the severity of OCD symptoms. In the present study, we utilized the clinician-administered semi-structured interview of the Y-BOCS at admission and discharge, and the self-report version after six weeks of treatment. Both versions were found to be interchangeable in a German sample [47]. The Y-BOCS is a 10-item-measure with five items designed to assess obsessional thoughts and five to assess compulsive behavior. Items are to be rated on a 5-point-scale ranging from 0 (no symptoms) to 4 (extreme symptoms). The German version of the Y-BOCS has shown good to very good inter-rater reliabilities, good internal consistency [48], and good convergent validity when compared to the Obsessive-Compulsive Inventory-Revised [49]. In the present study, the Y-BOCS displays good to excellent internal consistencies at all three assessment points (Cronbach’s *α =* .79 - .93).

#### Beck Depression Inventory-II

The BDI-II [45] is a widely used self-report questionnaire to measure depressive symptomatology within the last two weeks. The questionnaire consists of 21 items. For every item, participants have to choose one statement with an assigned value between 0 and 3. We used the German translation which exhibits satisfactory internal consistency, acceptable test-retest reliability, good convergent, discriminant and factorial validity, as well as good sensitivity to change [50–52]. Consistent with the findings by Hautzinger and colleagues [50], the internal consistencies in the present study varied from good to excellent at treatment intake and discharge (Cronbach’s *α =* .88 - .93).

#### Obsessive Beliefs Questionnaire

The OBQ [44] is a 44-item questionnaire designed to measure the key belief domains of OCD identified by the OCCWG. It consists of three factor-analytically derived subscales: (1) responsibility and threat estimation; (2) perfectionism and intolerance for uncertainty; (3) importance and control of thoughts (metacognitions). Answers are rated on a 7-point-scale (1 = “disagree very much” to 7 = “agree very much”) [44]. In the present study, we used the abbreviated German adaptation of the OBQ [53]. Factor analyses of the translated English version resulted in one version with only twenty-four items loading on the same factors as the English version. All scales of the German version of the OBQ exhibit good internal consistencies, acceptable retest reliabilities, and satisfactory convergent and discriminant construct validity as well as criterion validity [53]. As such, the psychometric quality of the German adaptation of the OBQ is comparable to the English original [53]. Consistent with the findings by Ertle and colleagues [53], the internal consistencies of the OBQ found in the present study varied from good to excellent at the three assessment points (Cronbach’s *α =* .92 - .93).

### Data analyses

To evaluate whether changes in obsessive beliefs during the first six weeks of treatment predict symptoms at discharge we conducted a hierarchical regression analysis. We entered obsessive-compulsive symptoms at intake of treatment in the first step and depressive symptoms at admission in the second step to control for potential confounding effects. Changes in obsessive beliefs between intake and six weeks of treatment were entered in the third step. To identify which specific obsessive belief domain best predicts treatment outcome, we conducted another hierarchical regression analysis. In this one, we also controlled for obsessive-compulsive and depressive symptom severity at intake (step 1 and 2) but entered changes in the three domains of the OBQ as predictors instead of the global score (step 3). We used SPSS version 23 for these analyses.

To examine whether changes in obsessive beliefs mediate changes in obsessive-compulsive symptoms, we used the procedure originally proposed by Baron and Kenny [54] and applied it to a multilevel framework as recently suggested [55]. For these analyses, we used the software R [56] with packages lme4 [57] and lmerTest [58]. The longitudinal nature of our design produced a multilevel data structure [55, 59], in which the lower level, or Level 1 data (i.e. the repeated measures of both obsessive-compulsive symptoms and obsessive beliefs), were nested within upper level, or Level 2, units (i.e. the participants). This data structure is appropriate for contemporary growth curve modeling techniques [60]. Given that pre-scores varied between the individuals, we examined this change over time in a multilevel random coefficient regression framework [61, 62]. According to the approach by Kenny, Korchmaros, & Bolger [63], mediation can be established if the following four statistical criteria are met: (1) Predictor and mediator are significantly related (path *a*), (2) predictor and criterion are significantly correlated (path *c*), (3) mediator and criterion are significantly related when the predictor is controlled for (path *b*), and (4) the relationship between predictor and criterion is smaller when the mediator is controlled for (path *c’*) compared to when it is not (path *c*). To examine whether obsessive beliefs mediate symptom changes, we tested a lower level mediation model [63], in which time (intake, after six weeks of treatment, discharge) was the predictor variable, obsessive-compulsive symptom severity the criterion, and obsessive beliefs the mediator. To control for potential confounding effects of changes in depressive symptom severity during treatment, we included changes in depressive symptoms during treatment as another predictor in each of the three regression analyses. The decision to control for (changes in) depressive symptom severity in our analyses was based on the high rate of depressive disorders in the sample of the present study. Moreover, it was based on prior research indicating confounding effects of (changes in) depressive symptoms both on treatment outcome [37, 64] as well as on the relation between changes in obsessive beliefs and obsessive-compulsive symptoms post-treatment [65]. Further potentially confounding variables such as gender, age, treatment duration, anti-depressive medication and comorbidities were neither related to (changes in) obsessive-compulsive symptoms nor to changes in obsessive beliefs (all *p*s > .05). Thus, we did not enter them as further covariates in our analyses. However, as individual means of the mediator may alter the estimation of paths in a multilevel framework [66], we included values of the mediator that were centered on the individual mean (group-mean centering) as well. We used Sobel’s test [67] to test the significance of the indirect effect of the hypothesized mediation model (i.e., the product of the regression weights from paths *a* and *b*). The test has already been discussed in a multilevel framework by Krull and MacKinnon [68]. For all our analyses, we set alpha at *p* < .05.

## Results

### Regression analyses

Results of the first hierarchical regression analysis are presented in Table 3. They indicate that - when controlling for obsessive compulsive symptom severity (*β* = .49, *t* = 4.77, *p* < .001) and depressive symptom load (*β* = .07, *t* = 0.73, *p* = .47) at intake - changes of obsessive beliefs (global score) between intake and after six weeks of treatment significantly predicted obsessive-compulsive symptoms at discharge (*β* = -.24, *t* = -2.38, *p* = .02) and explained 6 % of the outcome variance (Δ*F*(1,67) = 5.64, *p* = .02).

**Table 3:** Regression analysis with obsessive-compulsive symptoms (T3) as outcome and obsessive belief-changes (∆T1-T2) as predictor

	∆*R* ^*2*^	∆*F*	*p*	*B*	*SE B*	*β*	*t*	*p*
Step 1	.27	26.03	< .001					
Y-BOCS T1				0.66	0.13	.52	5.10	< .001
Step 2	.01	0.71	.40					
Y-BOCS T1				0.63	0.13	.50	4.77	< .001
BDI-II T1				0.06	0.08	.09	0.85	.40
Step 3	.06	5.64	.02					
Y-BOCS T1				0.61	0.13	.49	4.77	< .001
BDI-II T1				0.05	0.07	.07	0.73	.47
OBQ-Global ∆T1-T2				-1.89	0.80	-.24	-2.38	.02

Note. *N* = 71. Obsessive-compulsive and depressive symptoms (T1) were entered as covariates. T1 = intake, T2 = after six weeks of treatment, T3 = discharge. *Y-BOCS* = Yale-Brown Obsessive Compulsive Scale. *OBQ-Global* = Obsessive Beliefs Questionnaire Global Score. *BDI-II* = Beck Depression Inventory-II

As indicated in Table 4, the second hierarchical regression analysis showed that after controlling for variance explained by obsessive-compulsive and depressive symptom load at intake, entering all three belief domains in the next regression step revealed a clear tendency towards significance for the prediction of obsessive-compulsive symptoms at discharge (∆*F*(3,65) = 2.68, *p* = .05). All three belief domains together accounted for 8 % of the variance of outcome. Findings also indicated a trend for the domain “importance and control of thoughts” to independently predict treatment outcome (*β* = -.20, *t* = -1.74, *p* = .09).

**Table 4:** Multiple regression with obsessive-compulsive symptoms (T3) as outcome and belief domain-changes (∆T1-T2) as predictors

	∆*R*	∆*F*	*p*	*B*	*SE B*	*β*	*t*	*p*
Step 1	.27	26.03	< .001					
Y-BOCS T1				0.66	0.13	.52	5.10	< .001
Step 2	.01	0.71	.40					
Y-BOCS T1				0.63	0.13	.50	4.77	< .001
BDI-II T1				0.06	0.08	.09	0.85	.40
Step 3	.08	2.68	.05					
Y-BOCS T1				0.60	0.13	.48	4.46	< .001
BDI-II T1				0.08	0.08	.11	1.03	.31
OBQ-RES ∆T1-T2				0.34	0.68	.06	0.50	.62
OBQ-PER ∆T1-T2				-1.18	0.86	-.17	-1.37	.18
OBQ-IMP ∆T1-T2				-1.16	0.67	-.20	-1.74	.09

Note. *N* = 71. Obsessive-compulsive and depressive symptoms (T1) were entered as covariates. T1 = intake, T2 = after six weeks of treatment, T3 = discharge. *Y-BOCS* = Yale-Brown Obsessive Compulsive Scale. *OBQ* = Obsessive Beliefs Questionnaire. *RES* = responsibility and threat estimation. *PER* = perfectionism and intolerance for uncertainty. *IMP* = importance and control of thoughts (metacognitions). *BDI-II* = Beck Depression Inventory-II

### Mediation analyses

**Fig. 2:**
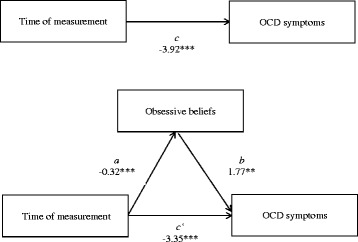
Mediation model with obsessive beliefs as mediator between time of measurement and obsessive-compulsive symptoms. *N* = 71. The unstandardized regression coefficients are presented. *p < .05, **p < .01, ***p < .001

Table 5 and Fig. 2 both show the results of the mediation analyses. When time was entered into the Level 1 regression equation predicting obsessive-compulsive symptom severity (path *c*) while controlling for changes in depressive symptom load between treatment intake and discharge, the regression coefficient indicated that obsessive-compulsive symptoms decreased significantly over the course of treatment (*B* = -3.92, *p* < .001). When time was entered into the Level 1 regression equation predicting obsessive beliefs (path *a*) while controlling for depressive symptom change, the regression coefficient showed that obsessive beliefs decreased significantly over time as well (*B* = -0.32, *p* < .001). When both time and depressive symptom change were controlled for, the regression coefficient showed that obsessive beliefs significantly predicted obsessive-compulsive symptoms (path *b*; *B* = 1.77, *p* = .001). This indicates that participants who showed fewer obsessive beliefs also suffered from a lower degree of obsessive-compulsive symptoms. Finally, when controlling for both obsessive beliefs and depressive symptom change, time still significantly predicted obsessive-compulsive symptoms (path *c*‘; *B* = -3.35, *p* < .001), but the regression coefficient was smaller than the one of path *c*. This is an indicator of a partial mediation. Sobel’s test of the indirect effect of time on obsessive-compulsive symptoms via obsessive beliefs was significant (*ab* = -0.57, *z* = -3.03, *p* = .002). Thus, all criteria for a partial mediation are met. This indicates that changes in obsessive-compulsive symptoms may be explained by changes in obsessive beliefs during inpatient treatment.

**Table 5:** Regression analyses for the hypothesized mediation model with obsessive beliefs as mediator

Step	Path	Predictor variable	Outcome variable	*B*	*SE B*	*t*	*p*
1	*c*	Time	Y-BOCS	-3.92	0.38	-10.41	< .001
2	*a*	Time	OBQ - Global	-0.32	0.05	-6.94	< .001
3	*b*	OBQ - Global	Y-BOCS	1.77	0.54	3.27	.001
	*c'*	Time	Y-BOCS	-3.35	0.40	-8.29	< .001

Note. *N* = 71. *Y-BOCS* = Yale-Brown Obsessive Compulsive Scale. *OBQ - Global* = Obsessive Beliefs Questionnaire Global Score

To examine whether treatment-associated changes in obsessive beliefs result both in changes in obsessions and compulsions, we computed two exploratory post-hoc mediational models: one with obsession severity as criterion and another one with compulsion severity as criterion while controlling for depressive symptom changes. Results show that changes in obsessive beliefs partially mediate the association between time and obsessions (*b*: *B* = 1.08, *p* < .001; *c*: *B* = -1.85, *p* < .001; *c’: B* = -1.51, *p* < .001; *ab* = -0.35, *z* = -3.19, *p* = .001), and between time and compulsions (*b*: *B* = 0.86, *p* = .008; *c*: *B* = -1.94, *p* < .001; *c’: B* = -1.66, *p* < .001; *ab* = -0.28, *z* = -2.55, *p* = .01). A comparison of the regression coefficients of the indirect effect of both models indicates that changes in obsessive beliefs explain changes in obsessions and compulsions to a similar extent. However, these findings should not be over-interpreted due to multiple testing and a potential alpha-error-accumulation.

## Discussion

The present study showed that changes in global obsessive beliefs during the first part of an intensive, primarily cognitive behavioral inpatient treatment for individuals with severe OCD predict treatment outcome. Further on, it offered preliminary evidence that changes in beliefs regarding the importance of thoughts and the need to control thoughts are most important in predicting treatment outcome when considering the belief domains identified by the OCCWG [27, 28]. Finally, it pointed out that changes in obsessive beliefs partially explain patients’ improvements during treatment and may thus constitute an essential change mechanism in intensive cognitive behavioral inpatient treatment for OCD.

Our findings are consistent with the large body of evidence demonstrating positive cross-sectional associations between obsessive beliefs and obsessive-compulsive symptom severity [31–34]. Moreover, they add to the findings that symptom changes during cognitive, behavioral and cognitive behavioral treatments are either predicted by the extent of obsessive beliefs at the beginning of treatment [35, 36] or even associated with [14, 35, 37–39] or predicted by [41] changes in obsessive beliefs during treatment. The finding that changes in obsessive beliefs *partially* mediated symptom changes is consistent with results showing that emotion activation and habituation explain symptom changes during CBT as well [69–71]. The non-significant trend of changes in metacognitions as a predictor of outcome is consistent with previous research showing that changes in metacognitions is the only obsessive belief domain that is associated with symptom changes during (C)BT [35, 41, 65]. Finally, our findings extend previous research as they show that changes in obsessive beliefs during an intensive inpatient CBT predict symptom changes longitudinally and even explain treatment success.

Thus, from a theoretical perspective, our results add weight to cognitive and meta-cognitive models of OCD that emphasize the role of dysfunctional (meta-cognitive) beliefs in the development and maintenance of OCD (see for example, [23–26]) and suggest that reductions in (metacognitive) obsessive beliefs lead to reductions in obsessive symptom severity. From a clinical perspective, they indicate that changes in obsessive beliefs are one of the working mechanisms of inpatient CBT in OCD. Thus, focusing more strongly on changing obsessive beliefs in CBT for OCD seems to be a promising treatment approach, especially as the extent of changes in obsessive beliefs in the present study was rather small; with a greater focus on obsessive beliefs, even greater symptom changes might be achieved.

Unfortunately, prior research does not unambiguously show which treatment is best suited to foster changes in obsessive beliefs. While it is clear that both CBT and ERP and cognitive therapy alone have the potential to decrease obsessive beliefs [14, 37, 72–74], findings regarding a potential superiority of either ERP or CT in changing dysfunctional beliefs are inconclusive. Whereas two studies show that ERP is sufficient to produce strong improvements in both obsessive-compulsive behavior and cognitions [36, 72], another study indicates that ERP is more effective if it is configured as a behavioral experiment rather than when it is accompanied by a habituation rationale [75]. Thus, at this point in time, it seems to be best practice to combine behavioral and cognitive elements in inpatient treatment for OCD (to enhance changes in obsessive beliefs) as recommended by current practice guidelines [8–10]. However, findings from the present study suggest focusing on potential changes in (metacognitive) obsessive beliefs in ERP debriefing to use the advantages of both behavioral and cognitive interventions.

Strengths of the present study consist in including a comparatively large number of inpatients with a validated diagnosis of OCD and in assessing obsessive-compulsive symptoms as well as obsessive beliefs at multiple assessment points. Limitations of the present study include the inpatient setting of the study and the lack of subgroup analyses for several OCD subtypes due to the limited sample size. The evaluation of a multimodal inpatient treatment concept without a waiting control group neither allows us to unambiguously conclude that symptom changes over time are due to treatment at all nor due to a specific treatment element. More specifically, our study does not provide any information about the exact effect of each treatment element on changes both in obsessive beliefs and obsessive-compulsive symptoms. Given that treatment consisted of both a disorder-specific individual and group therapy, and of a variety of additional therapies (occupational therapy, music therapy, sports therapy), we cannot specify which element is (most) responsible for changes in obsessive beliefs and obsessive-compulsive symptoms. Finally, our sample size precluded us from conducting analyses in OCD subgroups. This is unfortunate as specific dimensions of obsessive beliefs seem to be associated with specific types of OCD (e.g., elevated responsibility and threat estimation in the washing-/checking-subtype, elevated importance and control of thoughts in the checking-subtype, elevated perfectionism and uncertainty in the symmetry and ordering subtype; [31–34]). It might be that dimensions of obsessive beliefs that change during treatment (for example metacognitions) depend on the subtype of OCD the patients suffer from.

Future research should address these questions in controlled studies with larger sample sizes as these findings could have important implications for understanding variability in treatment response [76] and thus for improving treatment outcome. Given the multimodal treatment concept of the present study, future studies should assess and examine the specific contribution of stand-alone treatment elements in inpatient treatments on changes in obsessive beliefs and obsessive-compulsive symptoms. Moreover, future research should focus on investigating specific change mechanisms (i.e. habituation and changes in obsessive beliefs) in ERP and cognitive therapy in inpatient settings so that mediating effects can unambiguously be attributed to a specific treatment. Finally, future research should test whether (meta-)cognitive therapy enhances treatment outcome and thus is indicated in specific individuals and situations, for example in non-responders to behavioral therapy [77], in patients who primarily suffer from obsessive thoughts [78] or from strong belief distortions [79], and/or in patients with a strong aversion to exposure therapy [79].

## Conclusions

Changes in obsessive beliefs are one of the working mechanisms of multimodal, but primarily cognitive behavioral inpatient treatment in OCD. Thus, focusing more strongly on changing obsessive beliefs in (inpatient) CBT for OCD seems to be a promising treatment approach that might help further improve current treatments for individuals with OCD. Finally, it might represent an alternative treatment approach for non-responder to behavioral interventions and for those who suffer from strong cognitive biases or who reject exposure-based interventions.

## Abbreviations

BDI-II, beck depression inventory-II; CBT, cognitive behavioral therapy; DSM-IV, diagnostic and statistical manual of mental disorders-IV; ERP, exposure and response prevention; ICD-10, international classification of diseases-10; IMP, importance and control of thoughts (metacognitions); OBQ, obsessive beliefs questionnaire; OCCWG, obsessive compulsive cognitions working group; OCD, obsessive-compulsive disorder; PER, perfectionism and intolerance for uncertainty; RES, responsibility and threat estimation; Y-BOCS, yale-brown obsessive-compulsive scale
